# Altered glycosylation of glycodelin in endometrial carcinoma

**DOI:** 10.1038/s41374-020-0411-x

**Published:** 2020-03-23

**Authors:** Laura C. Hautala, Poh-Choo Pang, Aristotelis Antonopoulos, Annukka Pasanen, Cheuk-Lun Lee, Philip C. N. Chiu, William S. B. Yeung, Mikko Loukovaara, Ralf Bützow, Stuart M. Haslam, Anne Dell, Hannu Koistinen

**Affiliations:** 1 Department of Clinical Chemistry, University of Helsinki and Helsinki University Hospital, Helsinki, Finland; 2 Department of Life Sciences, Imperial College London, London, UK; 3 Department of Pathology, University of Helsinki and Helsinki University Hospital, Helsinki, Finland; 4 Department of Obstetrics and Gynaecology, The University of Hong Kong, Hong Kong SAR, China; 5 Department of Obstetrics and Gynecology, University of Helsinki and Helsinki University Hospital, Helsinki, Finland

**Keywords:** Diagnostic markers, Endometrial cancer, Mass spectrometry, Glycoconjugates, Immunohistochemistry

## Abstract

Glycodelin is a major glycoprotein expressed in reproductive tissues, like secretory and decidualized endometrium. It has several reproduction related functions that are dependent on specific glycosylation, but it has also been found to drive differentiation of endometrial carcinoma cells toward a less malignant phenotype. Here we aimed to elucidate whether the glycosylation and function of glycodelin is altered in endometrial carcinoma as compared with a normal endometrium. We carried out glycan structure analysis of glycodelin expressed in HEC-1B human endometrial carcinoma cells (HEC-1B Gd) by mass spectrometry glycomics strategies. Glycans of HEC-1B Gd were found to comprise a typical mixture of high-mannose, hybrid, and complex-type N-glycans, often containing undecorated LacNAc (Galβ1–4GlcNAc) antennae. However, several differences, as compared with previously reported glycan structures of normal human decidualized endometrium-derived glycodelin isoform, glycodelin-A (GdA), were also found. These included a lower level of sialylation and more abundant poly-LacNAc antennae, some of which are fucosylated. This allowed us to select lectins that showed different binding to these classes of glycodelin. Despite the differences in glycosylation between HEC-1B Gd and GdA, both showed similar inhibitory activity on trophoblast cell invasion and peripheral blood mononuclear cell proliferation. For the detection of cancer associated glycodelin, we established a novel in situ proximity-ligation based histochemical staining method using a specific glycodelin antibody and UEAI lectin. We found that the UEAI reactive glycodelin was abundant in endometrial carcinoma, but virtually absent in normal endometrial tissue even when glycodelin was strongly expressed. In conclusion, we established a histochemical staining method for the detection of endometrial carcinoma-associated glycodelin and showed that this specific glycodelin is exclusively expressed in cancer, not in normal endometrium. Similar methods can be used for studies of other glycoproteins.

## Abstract (web summary)

Glycodelin is a major endometrial glycoprotein. The authors analyzed glycan structures of endometrial carcinoma associated glycodelin and established a novel glycodelin-glycoform specific histochemical staining method. With this, they showed that glycodelin is differentially glycosylated in endometrial carcinoma tissue, as compared to normal endometrium, representing a neoantigen with potential clinical applications.

## Introduction

Glycodelin (also known as Progestagen Associated Endometrial Protein, PAEP, and Placental Protein 14, PP14) is a secreted human lipocalin-family protein mainly expressed in well-differentiated epithelial cells in human reproductive tissues, like endometrium and seminal vesicles [1, 2]. In premenopausal endometrium the expression of glycodelin is temporally regulated by progesterone, expression increasing shortly after ovulation until the end of the menstrual cycle. Depending on the cell of origin glycodelin is differently glycosylated, resulting in glycodelin isoforms with different biological actions [1, 3–8]. We have previously characterized four types of glycodelin, which are derived from different reproductive tissues: decidualized endometrium-derived glycodelin-A (GdA, isolated from amniotic fluid), glycodelin-F (isolated from follicular fluid), glycodelin-C (isolated from cumulus cells), and seminal vesicle-derived glycodelin-S (GdS, isolated from seminal plasma) [5, 7, 9, 10]. Each of these expresses a characteristic glycan profile. This translates to differences in functional assays. Thus, while glycodelin-A inhibits sperm–egg binding, i.e., fertilization, glycodelin-S prevents premature sperm capacitation, but does not prevent sperm–egg binding [10, 11]. In contrast, glycodelin-F prevents premature acrosome reaction of sperm, whilst glycodelin-C, which is believed to be derived from glycodelin-F via glycan processing by cumulus cells, stimulates sperm–egg binding [4, 12, 13]. Thus, it appears that different glycodelin isoforms participate in a cascade of activities regulating the sperm functions needed for successful fertilization. Furthermore, immunosuppressive activities of glycodelin have been ascribed to glycosylation [5, 14]. These include the inhibition of peripheral blood mononuclear cell (PBMC) proliferation [5, 15]. We have previously also reported that glycodelin-A inhibits trophoblast invasion, while deglycosylated glycodelin or differentially glycosylated glycodelin-F does not have this activity [16]. Tight regulation of trophoblast invasion is essential for successful pregnancy [17]. Based on these studies, glycodelin is considered as a remarkable example of how glycosylation may dictate the functions of glycoproteins [18].

We have suggested that glycodelin also acts as a tumor suppressor protein, at least in endometrial carcinoma, based on our findings showing that glycodelin transfection in HEC-1B human endometrial carcinoma cells cause morphological reversion from malignant phenotype to a more normal state [19–21]. This is mediated by a repressed PKC-δ activation [21]. Furthermore, in a preclinical mouse model, glycodelin-induced differentiation brought about reduced tumor growth [20]. Further proof for the inhibitory role of glycodelin in malignant transformation comes from the studies in which reversion of Ishikawa endometrial adenocarcinoma cells to resemble normal endometrial epithelial cells was induced by histone deacetylase inhibitors [22]. This differentiation was accompanied by glycodelin synthesis. Importantly, knockdown of glycodelin expression by RNA-interference blocked the malignant-to-normal reversion, demonstrating that glycodelin is crucial in this process. Glycodelin transfection into these cells caused reduced cell proliferation [23]. Malignant growth is associated with loss of cell differentiation, and many tumor suppressor genes that protect cells against malignant transformation are known to regulate cell differentiation [24]. Therefore it is not surprising that the expression of glycodelin is reduced in hormone-related cancers, including ovarian and breast cancers, and further reduced in less differentiated tumors [25–27].

Complex glycosylation, which greatly increases the structural diversity of glycoproteins, is essential for multicellular organisms [28]. Despite the utmost importance of protein glycosylation, the human glycome is not well characterized, mainly due to complexity of carbohydrate structures. Changes in protein glycosylation have been observed in several human cancers, where they affect tumorigenic properties, like cellular growth behavior, invasiveness and acquisition of metastatic potential [29–32]. Although many glycodelin isoforms have been characterized from reproductive tissues, nothing is known about the glycosylation of glycodelin produced by endometrial carcinoma tissue. Here, with the aid of glycomics analysis, we established a novel glycodelin–glycoform-specific histochemical staining method and showed that glycodelin is differentially glycosylated in endometrial carcinoma tissue as compared with normal endometrium.

## Material and methods

### Production and purification of glycodelin

HEC-1B cells (ATCC HTB-113), which do not normally produce glycodelin, were stably transfected with glycodelin cDNA [20, 21]. This cell line is a subline [33] of HEC-1 cells derived from a moderately differentiated papillary adenocarcinoma tumor from a patient with stage IA endometrial cancer [34]. The cells were cultured in RPMI-1640 (Lonza), supplemented with 10% fetal calf serum (FCS), 100 IU/ml penicillin, 100 µg/ml streptomycin and 2 mM l-glutamine, at 37 °C in humidified incubator with 5% CO_2_. The cell clones were authenticated as HEC-1B cells using microsatellite markers (Promega GenePrint 10 System) by the Institute for Molecular Medicine Finland (FIMM) Technology Centre, University of Helsinki.

Glycodelins from HEC-1B cell culture medium (HEC-1B Gd) and pooled amniotic fluids (glycodelin-A, GdA) were purified using a monoclonal anti-glycodelin antibody (F43-7F9) column as described previously [35]. Before purification Triton X-100 (0.1% final concentration) was added to the cell culture medium or amniotic fluid. After affinity purification, buffer was changed to PBS and any detached antibodies were removed using protein G column. Glycodelin preparations were concentrated using a centrifugal filter device (Amicon Ultra-4, 10,000 NMWL) and the concentrations were measured by an immunoassay [36].

### Glycomics analysis by MALDI-TOF/TOF and data interpretation

Glycomics analysis was performed using strategies previously optimized for GdA characterization [5, 7, 37]. Purified HEC-1B Gd (50 µg) was digested using sequencing grade trypsin (Promega). The N-glycans were then released by N-glycosidase F (Roche Applied Science) and separated on Sep-Pak C18 cartridges. The native N-glycans were permethylated using the sodium hydroxide procedure, subjected to chloroform extraction, and a further clean-up step using Sep-Pak C18 cartridges. The purified permethylated N-glycans were then dissolved in methanol and an aliquot of the sample was mixed with a 3,4-diaminobenzophenone matrix solution (10 mg/ml in 75% acetonitrile/water) (Acros Organics) at a 1:1 ratio (v/v). The glycan–matrix mixture was spotted on a MALDI plate and allowed to dry and crystallize. MALDI-TOF and -TOF/TOF (MS and MS/MS) data were obtained using a 4800 MALDI-TOF/TOF mass spectrometer (AB Sciex UK Limited) in positive ion mode. Argon was used as the collision gas with collision energy of 1 kV.

The MALDI MS and MS/MS data obtained were analyzed using Data Explorer 4.9 (AB Sciex UK Limited). Manual assignment of glycan sequences was based on the known biosynthetic pathways, with the aid of the glycobioinformatics tool, GlycoWorkBench [38].

### Lectin immunoassays

Lectin immunoassays were carried out essentially as previously described [36], using biotinylated lectins obtained from Vector Laboratories or from Sigma (Table 1). Briefly, biotinylated lectins were captured on streptavidin coated wells (96-well plate; PerkinElmer). In the assay, 25 µl sample (dilutions of HEC-1B Gd and GdA) and 200 µl assay buffer, supplemented with 1 mM CaCl_2_, MnCl_2_, and MgCl_2_, were incubated overnight at 4 °C in wells. After washing the wells Eu-labeled antibody against glycodelin (F25-9D8) was added for 2 h at room temperature. After washing enhancement solution was added and the fluorescence was measured.

**Table 1:** Lectin-immunoassay results.

Lectin^a^	Abbreviation	Reaction with^b^		Specificity^c^
		GdA	HEC-1B Gd	
*Sambucus nigra*	SNA	+++	−	Neu5Acα6Gal/GalNAc
*Erythrina cristagalli*	ECL	−	+++	Galβ4GlcNAc
*Ulex europaeus* I	UEAI	−	+++	αFuc
Concanavalin A	Con-A	+	+++	αMan, αGlc
*Pisum sativum*	PSA	+	+	αMan, αGlc
*Lens culinaris*	LCA	+	+++	αMan, αGlc
*Ricinus communis* I	RCA I	+	+	Gal
*Griffonia simplicifolia* II	GSL II	−	−	αGal
*Griffonia simplicifolia* I	GSL I	−	−	αGal, αGalNAc
*Dolichos biflorus*	DBA	−	−	αGalNAc
Soybean	SBA	+	+++	α > βGalNAc
*Sophora japonica*	SJA	−	−	βGalNAc
*Vicia villosa*	VVA	−	−	GalNAc
*Wistera floribunda*	WFA	+	++	GalNAc
Wheat Germ	WGA	+	++	GlcNAc
Succinylated Wheat Germ	WGA_succ_	+	+++	GlcNAc
*Datura stramonium*	DSL	−	−	(GlcNAc)_2–4_
*Lycopersicon esculentum*^d^	LEL	−	−	(GlcNAc)_2–4_
*Solanum tuberosum*	STL	+	+	(GlcNAc)_2–4_
Peanut	PNA	−	−	Galβ3GalNAc
Jacalin	Jacalin	−	−	Galβ3GalNAc
*Phaseolus vulgaris* Erythroagglutinin	PHA-E	−	−	Galβ4GlcNAcβ2Manα6 (GlcNAcβ4) (GlcNAcβ4Manα3) Manβ4
*Phaseolus vulgaris* Leucoagglutinin	PHA-L	+	+	Galβ4GlcNAcβ6(GlcNAc β2Manα3)Manα3

^a^Lectin from *Wisteria floribunda* (WFA, L-1516) was obtained from Sigma. Other lectins were from Vector Laboratories as Biotinylated Lectin Kits I, II, and III.

^b^−, no reactivity (signal <1.5-fold of that of blank, i.e., signal obtained with biotinylated lectin and labeled antibody without GdA or HEC-1B Gd); +, positive signal (>1.5-fold blank); ++, >2- and <4-fold difference between the reactivity with GdA and HEC-1B Gd (after deduction of blank value); +++, >4-fold difference between the reactivity with GdA and HEC-1B Gd. The relative reactivity between different lectins cannot be deduced from the table.

^c^Specificity is based on the manufacturer’s (Vector Laboratories) information.

^d^High background signal (perhaps due to direct binding of the detection antibody to the lectin).

### Invasion assays

Human placental tissue was obtained with a written consent from women who underwent surgical termination of pregnancy in the first trimester. Primary trophoblast expressing CK7 and HLAG were obtained as described [39, 40]. Briefly, the placental villi were digested with trypsin and DNAase, filtered through 100 and 40 μm filters, and centrifuged by using density gradient media (Ficoll-Paque). The isolated cells were then incubated in a plastic culture dish for 20 min to remove adherent leukocytes. Nonadherent cells were seeded onto fibronectin-coated plates and incubated overnight.

The adherent primary trophoblasts and JEG-3 human choriocarcinoma cells (ATCC HTB-36) were cultured in Dulbecco’s Modified Eagle Medium and Eagle’s Minimum Essential Medium (EMEM) (Lonza), respectively, supplemented with 10% FCS, 100 IU/ml penicillin, 100 µg/ml streptomycin, and 2 mM l-glutamine, at 37 °C in a humidified, CO_2_-controlled (5%) incubator. Primary trophoblasts (3 × 10^5^) and JEG-3 cells (1.5 × 10^5^) in serum free medium containing 0 or 10 µg/ml either GdA or HEC-1B Gd were added into Polycarbonate Membrane Transwell® inserts with 8.0 µm pore size (Corning, Inc.) covered with 100 µl phenol-red free Matrigel^TM^ Basement Membrane Matrix (BD Biosciences) diluted 1:2 with EMEM. The inserts were placed into 24-well plate containing 400 µl of culture medium with 10% FCS and incubated for 22 h. The Matrigel and the non-invasive cells were removed using cotton swabs and the membranes were stained with 0.09% crystal violet for 10 min. The cells were counted under a microscope and the mean of three fields per membrane was calculated.

### XTT cell viability assays

Human female peripheral blood was obtained from the Hong Kong Red Cross. PBMCs were isolated by density gradient centrifugation (Ficoll-Paque). Cell viability was determined by the XTT assay (Roche Diagnostics) according to the manufacturer’s protocol. In brief, PBMCs (3 × 10^4^) were incubated with 0.01, 0.1, 1, and 10 µg/ml glycodelins for 36 h, and XTT-labeling mixture was added 12 h before absorbance measurement at 450 and 595 nm. The cell viability was expressed as relative viability (%) using the following equation:

$${\mathrm{Relative}} \,\, {\mathrm{viability}} \,\, (\%) = \,\, 	({\mathrm{Abs}} \,\, {\mathrm{GdA}} - {\mathrm{Abs}} \,\, {\mathrm{blank}}) / \\ 	({\mathrm{Abs}} \,\, {\mathrm{control}} - {\mathrm{Abs}} \,\, {\mathrm{blank}}) \times 100\%.$$

### Histochemical stainings

Formalin-fixed, paraffin-embedded tissue samples were collected at Pathology Department, the Helsinki University Hospital, Helsinki, Finland. Tissue microarrays (TMA) including 75 endometrial carcinomas of the endometrioid subtype and 52 samples of normal control endometrium were prepared as described previously [41]. The clinicopathological data of the patients selected for this study are shown in Table 2. Three secretory phase endometrium samples served as positive controls. Approvals of the Institutional Review Board of the Helsinki University Hospital and the National Supervisory Authority for Welfare and Health were obtained.

**Table 2:** Clinicopathological data in relation to glycodelin (Gd), UEAI lectin (UEAI) and UEAI-glycodelin (UEAI-Gd) staining.

	*n* (%)^b^	Gd pos. *n* (%)^c^	UEAI pos. *n* (%)^c,d^	UEAI-Gd pos *n* (%)^c^
**Endometrial carcinoma**^a^	75 (100)	22 (29)	57 (77)	36 (48)
Grade				
1–2	65 (87)	21 (32)	51 (78)	33 (51)
3	10 (13)	1 (10)	6 (67)	3 (30)
*p* value^e^		*p* = 0.26	*p* = 0.42	*p* = 0.31
Stage				
I	62 (83)	18 (29)	48 (79)	29 (47)
II–IV	13 (17)	4 (31)	9 (69)	7 (54)
*p* value^e^		*p* = 1.0	*p* = 0.48	*p* = 0.76
Tumor size^f^				
Diameter < 2 cm	22 (30)	7 (32)	16 (73)	11 (50)
Diameter ≥ 2 cm	51 (70)	14 (27)	39 (78)	24 (47)
*p* value^e^		*p* = 0.70	*p* = 0.49	*p* = 0.69
LVI^f^				
Yes	17 (24)	6 (35)	12 (71)	7 (41)
No	55 (76)	15 (27)	43 (80)	27 (49)
*p* value^e^		*p* = 0.80	*p* = 0.86	*p* = 1.0
**Normal endometrium**^a^	52 (100)	6 (12)	17 (34)	1 (2)
*p* value (cancer vs normal)^e^		*p* = 0.018	*p* < 0.0001	*p* < 0.0001

^a^The mean ± SD age of patients was 65.7 ± 11.3 years (range 37–92 years) and for controls 57.9 ± 6.9 years (range 38–77).

^b^Number of samples in each category and in parenthesis percent.

^c^Number and in parenthesis percent of positively stained samples in each category.

^d^In UEAI staining one endometrioid carcinoma (grade 3, stage I, diameter ≥ 2 cm and no LVI) and two control samples were missing.

^e^*p* values (Fisher’s exact test, two-sided) between frequency of positively stained samples in different subgroups or between carcinoma and normal endometrium.

^f^Tumor size or lymphovascular invasion (LVI) were not available from 2 or 3 patients, respectively.

Five µm thick sections were cut from the tissue and TMA blocks and placed onto glass slides. For histochemical stainings (below), the sections were deparaffinized and rehydrated. Stainings were scored by an experienced pathologist (A.P.).

### Glycodelin staining

To enhance the antigen retrieval the sections were heated in citrate buffer (pH 6.0) in a microwave oven for 10 min. This was followed by the addition of peroxidase-blocking solution (Dako REAL, #S2023, DAKO) for 30 min and horse serum (diluted 1:20 in PBS, Vector Laboratories) for 20 min, both at room temperature. Rabbit (#1) anti-glycodelin IgG [25] was used as the primary antibody (3 µg/ml, incubated for overnight at +4 °C) and the IgG fraction from preimmune serum of the same rabbit (3 µg/ml) was used as a negative control. Labeled polymer-HRP anti-rabbit (DAKO, K4009) was used as a secondary detection reagent (30 min at room temperature). Histofine Simple Stain AEC Solution (Nicherei Biosciences) was used as a chromogen for antigen detection. Counterstaining was performed with modified Mayer’s staining solution (#01820, Histolab Products Ab). For the progesterone receptor (PR) staining, 3 µm sections were stained with Ventana Benchmark Ultra (Roche/Ventana). Heat-induced epitope retrieval was performed using standard pretreatment buffer CC1 (Roche/Ventana), 64 min/98 °C. The slides were incubated with PR antibody (dilution 1:50, clone 16, NCL-L-PGR-312, Novocastra) 32 min/37 °C. For detection we used multimer-based detection kit Optiview (760-700, Roche/Ventana). The slides were counterstained with Mayer’s hematoxylin, and were then dehydrated and mounted for viewing with microscope. Glycodelin staining was scored either strong positive, weak/focal positive, or negative. PR staining was scored either positive or negative.

### Glycodelin–UEAI-lectin in situ proximity ligation assay (PLA®)

Unless otherwise indicated, in situ PLA® was performed using the Duolink® reagents essentially according to manufacturer’s instructions (Sigma-Aldrich). Briefly, endogenous peroxidase activity was blocked by 30 min treatment with 0.3% H_2_O_2_ (DUO82054) at room temperature. Tissue slices were blocked using blocking solution (DUO82007) for 30 min at 37 °C. Monoclonal mouse antibody against glycodelin [3 µg/ml, F25-9D8 [36]] and biotinylated *Ulex europaeus* agglutinin I (UEAI) (5 µg/ml, Vector Laboratories) were diluted in antibody diluent (DUO82008) and incubated with sections overnight at 37 °C. Duolink in situ PLA Probe Anti-Mouse PLUS (DUO92001) and streptavidin (2 µg/ml) conjugated with Duolink In Situ probemaker MINUS (DUO92010) were used as detection probes. The reaction with probes, ligation, amplification (2 h at 37 °C), and detection were performed according to the manufacturer’s instructions. The luminal and cytoplasmic staining were scored either positive or negative.

### Lectin staining

Endogenous peroxidase activity was blocked using peroxidase-blocking solution (Dako REAL, #S2023) for 30 min, followed by incubation with horse serum (diluted 1:20 in PBS, Vector Laboratories) for 20 min, both at room temperature. Biotinylated UEAI lectin (25 µl/ml in PBS containing 1% BSA, 1 mM CaCl_2_, 1 mM MgCl_2_, 1 mM MnCl_2_) was incubated overnight at +4 °C. ABC kit (PK-6100, Vector Laboratories) was used as a secondary detection reagent according to manufacturer’s instructions. Staining with AEC and Mayer’s staining solution were performed as in glycodelin staining. Staining in carcinoma cells and epithelial cells of normal endometrium was scored either positive or negative (<10% of cells stained). Blood vessels were not included in the evaluation.

### Data analyses

Fisher’s exact test (two-sided, IBM SPSS Statistics 24) was used to evaluate associations between different patient groups and staining. *T*-test (two-sample unequal variance, two-sided, Microsoft Excel) was used to evaluate differences in biological activity of different glycodelin preparations.

## Results

### Glycomics analysis of glycodelin expressed by HEC-1B cells (HEC-1B Gd)

**Fig. 1:**
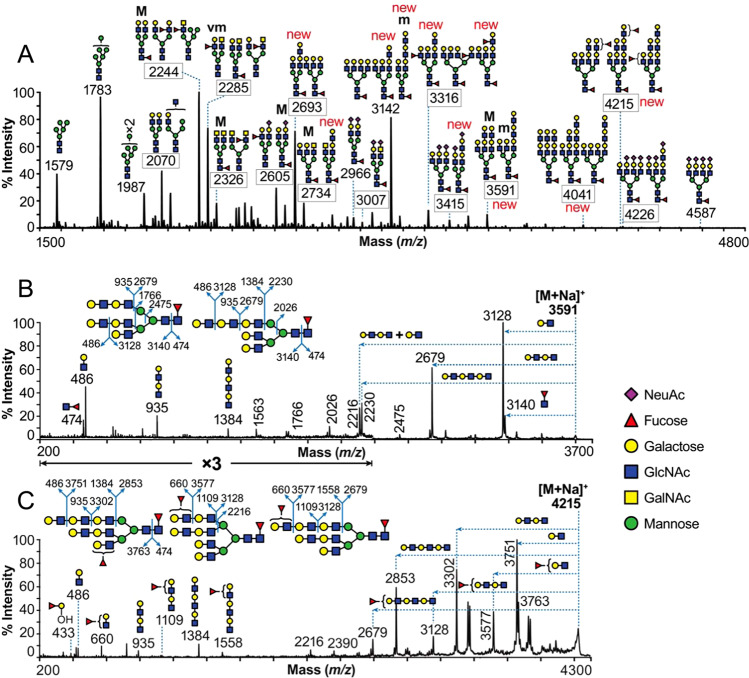
Glycomics analysis of glycodelin expressed by HEC-1B cells (HEC-1B Gd). **a** MALDI-TOF mass spectra of permethylated N-glycans from HEC-1B Gd. “new” above particular structure, or next to a *m/z* value, indicates new structure or a set of structures when compared with the N-glycome of GdA analyzed before [5]. All signals observed are singly charged sodiated ions [M + Na]^+^, and their structural assignment is based on monosaccharide composition, MS/MS fragmentation analyses (boxed *m/z* values) and knowledge of the glycan biosynthetic pathways. Fully annotated spectra that have been expanded on the *m/z* axis to enable all components to be visualized, are presented in Supplementary Fig. Sugars shown on top of a bracket have not had their antenna location unequivocally defined. For simplicity, one branching pattern for tri-antennary structures is shown. Therefore, the position of an antenna in a cartoon does not imply designation of a specific arm. “M”, “m” and “vm” designations indicate major, minor and very minor abundances, respectively. **b**, **c** Evidence for LacNAc extension of N-glycans. MALDI-TOF/TOF MS/MS of the molecular ion at (**b**) *m/z* 3591 and (**c**) 4215 as detected on the MALDI-TOF MS spectrum (see **a**). The fragment ions are consistent with the sequences shown in corresponding insets (major abundance structures). The horizontal arrows on the spectra indicate losses of glycan moieties from the molecular ion. **c** The fucose residues outside the bracket have not had their location unequivocally defined.

Glycomics analysis was performed using strategies previously optimized for GdA characterization [5, 7]. The permethylated N-glycans were subjected to MALDI-MS profiling and MALDI-MS/MS sequencing. The complete MALDI-MS spectrum of the N-glycans of HEC-1B Gd and annotation of the most abundant informative molecular ions is shown in Fig. 1a. Comprehensive annotations are shown on the magnified spectrum in Supplementary Fig. The glycan structures were manually assigned by combining information on the N-glycan compositions and knowledge of human N-glycan biosynthetic pathways. These assignments were then further confirmed and/or corrected based on MS/MS data, which were also manually interpreted. For practical reasons not all molecular ions in the spectra could be sequenced by MALDI-TOF/TOF MS/MS. Nonetheless it was possible to sequence representative members of each family of glycans as well as molecular ions not previously reported in GdA. Molecular ions selected for MS/MS are indicated with boxed *m/z* values in Supplementary Fig.

Overall, the HEC-1B Gd glycan population comprised of a mixture of high-mannose, hybrid, truncated, and abundant complex N-glycans that is typical of other female glycodelins (Fig. 1a). The most abundant (in terms of relative abundance) complex N-glycans corresponded mainly to core-fucosylated structures with undecorated Galβ1–4GlcNAc (LacNAc) units on their antennae (*m/z* 2244, 2693, 3142, 3591, and 4041). N-glycans with undecorated GalNAcβ1–4GlcNAc (LacdiNAc) units, also mainly core-fucosylated, were also detected (*m/z* 2285, 2326 and 2734), but in minor abundance. Both the LacNAc and LacdiNAc containing N-glycans were occasionally decorated by NeuAc (*m/z* 2605, 2966, 3007, 3415, 4226, and 4587) or fucose (*m/z* 2244, 2285, 2326, 2605, 3316, and 4215) residues. Comprehensive analysis of the molecular ions, the composition of which corresponded to multiples of LacNAc units (e.g., *m/z* 3142, 3316, 3415, 3591, 4041, and 4215), indicated that they were actually a mixture of isomers of which a minority contained linear poly-LacNAc antennae. Two examples are the structures detected for the molecular ions at *m/z* 3591 and 4215. MALDI-TOF/TOF MS/MS of the former molecular ion exhibited fragment ions that corresponded to losses of two and three LacNAc repeating units (*m/z* 2679 and 2230, respectively, Fig. 1b) indicating the presence of linear poly-LacNAc units. Similarly, the molecular ion at the *m/z* 4215 exhibited fragment ions that corresponded to losses of two and three LacNAc repeating units, each further modified with a fucose residue (*m/z* 3128 and 2679, respectively, Fig. 1c). The detection of the fragment ions at *m/z* 433 and 660, indicated that in certain cases the fucose residue was at the non-reducing terminus of the above poly-LacNAc units. Particularly, the presence of fragment ion at *m/z* 433, together with the absence of a fragment ion at *m/z* 834 (indicating the absence of Le^b/y^ epitopes for the specific molecular ion), suggested the presence of H type antigen (Fucα1–2Galβ1–3/4GlcNAcβ).

As compared with previously reported N-glycans of GdA [5, 7], the above N-glycan population of HEC-1B Gd exhibited differences with the most noticeable being the following: (i) the reduced abundance of Sda (NeuAcα2-3[GalNAcβ1-4]Gal) epitope (detected only at *m/z* 2646, Supplementary Fig.); (ii) the reduced abundance of sialylated N-glycans; and (iii) the increased abundance of linear poly-LacNAc units that were further modified with NeuAc or fucose residues.

### Reactivity with lectins

The glycan-analyses data allowed us to select lectins that potentially show differential binding to GdA and HEC-1B Gd. Also, several other lectins were tested (Table 1). The lecting binding studies confirmed the differences between the glycans of HEC-1B Gd and GdA. We found that UEAI and *Erythrina cristagalli* agglutinin (ECL) exclusively recognized HEC-1B Gd, not GdA, while *Sambucus nigra* agglutinin (SNA) recognized only GdA. Several other lectins bound preferentially to HEC-1B Gd but bound weakly also to GdA.

### Bioactivity of HEC-1B Gd

**Fig. 2:**
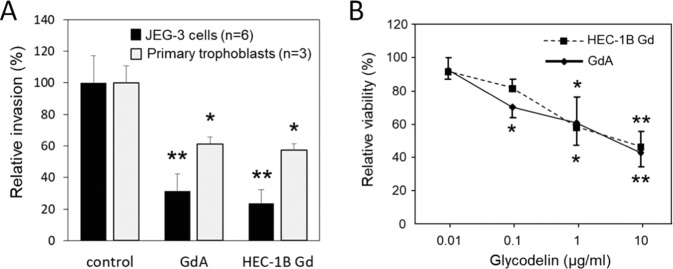
Biological activity of HEC-1B Gd and GdA. **a** Trophoblast invasion assays were performed using primary trophoblasts (*n* = 3) and JEG-3 choriocarcinoma cells (*n* = 6). **b** Peripheral blood mononuclear cell (PBMC) viability was evaluated by XTT assay (*n* = 4). *p < 0.05 or **p < 0.01 vs. control (*T*-test). Data represent mean ± SE.

The bioactivity of HEC-1B Gd was studied by assays in which we previously have found glycosylation to be important for the activity of glycodelin [5, 7, 16]. Trophoblast invasion assays were conducted both using primary trophoblast cells and JEG-3 choriocarcinoma cells. Irrespective of the differential glycosylation of HEC-1B Gd and GdA, these glycodelin isoforms had similar inhibitory activity on trophoblast cell invasion (Fig. 2a). The same was observed in inhibition of PBMC viability (Fig. 2b).

### Histochemical staining of glycodelin and its UEAI reactive glycoform in endometrial carcinoma tissue

To test whether the differences found in endometrial carcinoma cell line (HEC-1B Gd) and endometrium (GdA) derived glycodelins are also found in endometrium tissue of cancer patients and controls, we established a specific staining method for UEAI lectin-reactive glycodelin (UEAI–glycodelin).

**Fig. 3:**
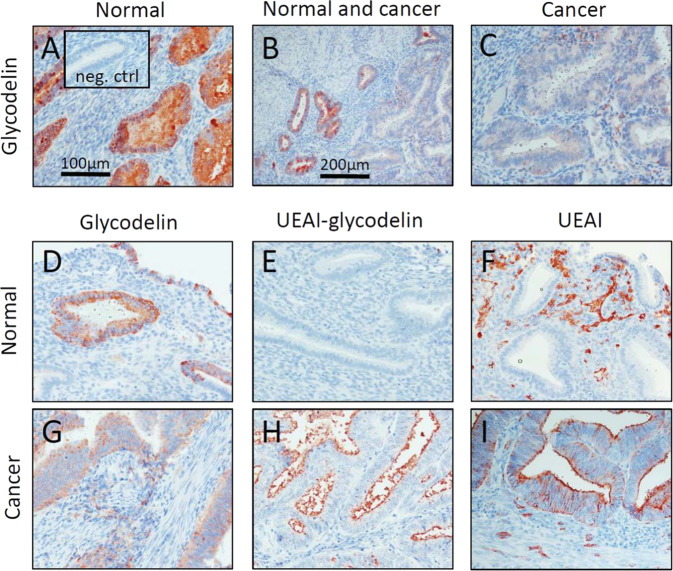
Histochemical staining of glycodelin, UEAI lectin–reactive glycodelin (UEAI–glycodelin) and UEAI–binding glycans in normal endometrium and endometrial carcinoma tissues. Example of glycodelin staining in a whole tissue section (**b**), which has both cancerous region (**c**) and normal secretory endometrium (**a**). Insert in **a** is a negative control in which the glycodelin antibody has been replaced with preimmune IgG. Representative examples of strong glycodelin staining (with antibody) (**d**, **g**), UEAI–glycodelin staining (proximity-ligation based detection with glycodelin antibody and UEAI lectin) (**e**, **h**) and staining of UEAI lectin-reactive glycans (UEAI lectin staining) (**f**, **i**) in normal endometrium (**d**–**f**) and endometrial carcinoma (**g**–**i**) TMA samples. Scale in **a** is 100 µm and the same scale applies to **c**–**i**. Scale in **b** is 200 µm.

First, we stained glycodelin with an antibody that does not discriminate between the glycoforms. In TMA, the amount of samples with strong glycodelin staining was not different between the endometrial carcinoma (5.3%) and control groups (5.8%) (*p* = 1.0, Fisher’s exact test), while the overall glycodelin staining (i.e., both strong and weak focal staining) was more frequent in carcinoma group (29.3% vs. 11.5%, *p* = 0.018) (Fig. 3, Table 2). In histologically normal endometrium, glycodelin staining was found in glandular epithelium, while in tumoral tissue glycodelin was localized in the carcinoma cells (Fig. 3a–d, g). However, even the strong staining in the carcinoma samples tended to be weaker and more focal than that in non-malignant endometrial tissue, including three histological sections of the secretory phase endometrium, which showed very strong glycodelin staining (Fig. 3a). Interestingly, three endometrial samples in which areas of both cancerous and histologically normal endometrium were present showed strong glycodelin staining only in non-malignant area, while cancerous regions in the same sections were glycodelin negative or only weakly stained (Fig. 3a–c).

In in situ proximity ligation based staining of UEAI lectin-reactive glycodelin (UEAI–glycodelin) 30.7% of the endometrioid carcinoma tissues showed luminal staining, 1.3% cytoplasmic staining, and 16.0% both cytoplasmic and luminal staining (Fig. 3e, h, Table 2). The non-cancerous tissues showed positive staining only in one case (*p* < 0.001 between endometrioid carcinoma and control samples).

When the tissue sections were stained with UEAI lectin alone, blood vessels were found to be strongly positive both in normal and cancerous tissues, while epithelial cells were more often positive in cancer samples (Fig. 3f, i). The staining in cancer was also stronger and more often found in cytoplasm. All the UEAI–glycodelin positive cancer samples were also UEAI-staining positive.

Almost all (86.5%) carcinoma samples were PR positive and the frequency of PR positive samples was similar irrespective of whether the samples were glycodelin or UEAI–glycodelin positive or negative (*p* > 0.26 for both). In the endometrioid carcinoma samples, frequencies of glycodelin, UEAI–glycodelin, and UEAI-staining positive samples were not statistically different between low (grades 1 and 2) and high (grade 3) grade endometrioid tumors (Table 2). Likewise, no differences in the frequency of positively-stained samples were found between small (tumor diameter < 2 cm) and large tumors (diameter ≥ 2 cm) or whether lymphovascular invasion was observed (for all *p* > 0.49). Local carcinomas (stage I) were not different, in respect of staining frequencies, from those showing spread beyond the uterine corpus (stages II–IV).

## Discussion

Glycodelin represents one of the foremost examples on how glycosylation dictates the function of a glycoprotein [1, 2, 8, 10, 18]. Although protein glycosylation is often altered in cancer [29–32] nothing is known about the glycosylation of glycodelin in endometrial carcinoma. Here, we describe a novel method for histochemical staining of a specific glycodelin–glycoform, based on in situ proximity ligation with a lectin and antibody. With this method we showed that glycodelin is differentially glycosylated in endometrial carcinoma, as compared with normal endometrium.

In order to establish a specific histochemical staining method for glycodelin expressed in endometrial carcinoma, we first carried out glycomics analysis of glycodelin expressed in the human endometrial carcinoma cell line HEC-1B. Our aim was to ascertain how the glycomic profiles differed from those reported for first trimester GdA and to use that information to support the selection of lectins for further studies. To this end, glycomics analysis showed that the major N-glycans of HEC-1B Gd exhibited decreased abundance of sialic acid (NeuAc) residues and carried only very low levels of Sda containing glycans (none in the high-mass region), abundantly expressed in GdA, especially on larger glycans [7]. On the contrary, HEC-1B Gd N-glycans exhibited increased abundance of linear poly-LacNAc units (two or three LacNAcs) on their antennae, which were mainly undecorated, or to a lesser extent further modified by a NeuAc or fucose residue. This is further supported by the report showing that β1–4-galactosyltransferase, which is involved in poly-LacNAc synthesis, is increased in endometrial cancer [42]. These differences are not likely to be explained by variations observed in GdA preparations isolated from endometrium and amniotic fluid of different individuals and during different times of pregnancy [43].

Next, we tested lectins that, based on the above glycomics analysis, were predicted to differentiate between carcinoma associated HEC-1B Gd and normal endometrium GdA. Decreased sialylation of HEC-1B Gd as compared with GdA suggested that SNA may differentiate between these two glycodelins and, indeed, SNA did not bind to HEC-1B Gd, while strong reaction with GdA was observed. Similarly, more abundant LacNAc epitopes in HEC-1B Gd agreed with increased ECL binding. However, it was somewhat surprising that LEL, DSL, and STL lectins, which should also bind poly-LacNAc [44], did not react well with HEC-1B Gd or GdA. Negative results are not necessarily informative as they may reflect low-binding affinity of the lectin in the assay conditions, rather than indicate genuine lack of the given carbohydrate epitope. Moreover, as the lectin binding was determined with a sandwich type of an assay, using glycodelin-specific monoclonal antibody and lectins, we could not rule out possible sterical hindrance preventing the binding of both the antibody and some of the lectins.

UEAI, a lectin that has been found to strongly recognize H type epitopes [45, 46], was found to bind well HEC-1B Gd. Indeed, MALDI-TOF/TOF MS/MS analysis showed a minor marker fragment ion corresponding to a fucose residue attached to a terminal galactose residue (*m/z* 433, Fig. 1c), suggesting the presence of H type antigen on elongated LacNAc antennae on the HEC-1B Gd. UEAI lectin also reacts with other fucosylated epitopes, such as Le^y^ and Le^x^ epitopes [45–47]. However, UEAI did not show any significant binding to GdA. Since GdA exhibits Le^x^ antigens [9], binding to it should have been expected to some extent [5, 7, 9]. As fucosylated glycans in GdA are more prevalent in the Asn-63 glycosylation site [9], it is possible that the binding of UEAI to this glycosylation site could be blocked by the antibody binding in close proximity.

We have previously characterized several glycodelin isoforms, which differ especially in the level of sialylation and abundance of glycans with Sda epitopes [5, 7, 9], and have different effects on the viability of PBMCs. This may be due to reduced sialic acid content, as desialylation of GdA was found to abolish the immunomodulatory activity. Thus, it was somewhat surprising that HEC-1B Gd, which also appears to contain less sialic acids than GdA, had inhibitory activity on PBMC viability similar to GdA. However, as several different glycans in GdA are sialylated, it is possible that only some of those, which are also present in HEC-1B Gd, mediate the antiproliferative effect of glycodelin on PBMC or that different glycans can produce similar effect. Using enzymatic deglycosylation and/or differentially glycosylated glycodelin isoforms, we have found, that in addition to PBMC proliferation, the inhibitory activity of GdA on trophoblast invasion is dependent on glycosylation [16]. Again, despite the differences in glycosylation between GdA and HEC-1B Gd, both of these glycodelin glycoforms inhibited trophoblast invasion in similar potency.

After establishing, by glycan-analyses and lectin-binding studies, that HEC-1B endometrial carcinoma cells produce glycodelin with different N-glycans than normal endometrium, we studied whether this can be seen also in endometrial carcinoma tissues. This is feasible as glycosylation of endometrial proteins is not only dependent on the phase of the menstrual cycle and menopausal status, but is further changed in malignant endometrium [48]. First, we studied glycodelin expression in normal endometrium and endometrial carcinoma. Previous studies have shown that high glycodelin expression is restricted to secretory endometrium, while expression in proliferative phase or postmenopausal endometrium is very low if detectable at all [1]. In order to detect also low levels of glycodelin, we used high antibody concentration, which stained normal secretory endometrium very strongly. Immunoreactive glycodelin was not often found in TMA samples, mostly obtained from postmenopausal women, and, apart from the few TMA samples representing normal secretory endometrium, the staining was weaker than that observed in control histological sections of normal secretory endometrium. Relatively strong glycodelin immunoreactivity was found in some samples, but there was no difference in frequencies of such samples between normal and endometrioid carcinoma groups. However, weak and focal glycodelin staining was observed more frequently in carcinoma samples. Interestingly, in few carcinoma samples containing adjacent histologically normal secretory epithelium, strong staining was observed only in the latter, suggesting that glycodelin expression may be reduced during malignant transformation. Previously most studies have not found glycodelin in cancerous endometrium [1]. One group has reported frequent glycodelin staining in endometrial cancer, which appears to be somewhat dependent on the used antibody [49]. Of note, glycodelin transcripts (mRNA) are more abundant in normal uterus than in uterine carcinomas [IST Online® ver. 2.1.3 database, containing gene expression data of about 20,000 samples, http://ist.medisapiens.com/ [50]]. Glycodelin is also expressed in ovarian and breast cancers, where the levels are reduced in less differentiated tumors [25–27].

We found more frequent and stronger UEAI staining in endometrial cancer than in normal endometrium. Previous lectin studies have also shown that the binding of UEAI and its glycan epitope, H type antigen, are increased in endometrial carcinoma [51–53]. This corroborates with other reports showing that in endometrial carcinoma there is an increased expression of fucosyltransferases responsible for the expression of fucosylated epitopes including H type antigen [54, 55]. Also, lectin histochemistry on breast cancer tissues has shown a major reduction of UEAI staining after endo-β-galactosidase digestion, suggesting that UEAI lectin on breast cancer tissues recognizes fucosylated antigens on elongated LacNAc antennae of N-glycans [56].

As the UEAI lectin binds HEC-1B Gd, but not GdA, and UEAI staining was stronger in endometrium carcinoma than in normal tissue, we next studied whether the specific UEAI reactive glycodelin (UEAI–glycodelin) would be more abundant in endometrial carcinoma than in normal endometrium. To this end, we established an in situ proximity ligation assay [57] with a combination of glycodelin antibody and UEAI lectin. A similar combination of an antibody and a lectin has previously been used for proximity-ligation assay for serum and tissue lysate samples [58] and an in situ approach has been used with a combination of protein- and glycan-specific antibodies [59, 60]. However, to our knowledge this is the first report for such a protein-glycoform-specific histochemical staining with a lectin-antibody combination.

UEAI–glycodelin staining was almost exclusively found in endometrial carcinoma tissues and it was more frequent than the glycodelin staining. This is in keeping with the strong binding of UEAI to HEC-1B Gd, while no significant binding to GdA was observed. The staining was mostly found in luminal part, which suggests that the UEAI reactive glycans are mature only at the late stage of the secretory pathway. As proximity-ligation is a very sensitive technique, all glycodelin positive samples would have been expected to be positive also in UEAI–glycodelin staining. However, this was not the case. Thus, it is possible that in carcinoma samples glycodelin does not always carry UEAI reactive glycans. It has to be also noted that the tissue cores in TMA were quite small and the stained sections were not consecutive. Therefore, the pairwise comparison may not tell the whole truth. The lack of the glycodelin staining in some of the UEAI–glycodelin positive samples may be related to the same fact and a less sensitive method. Another possibility is the lack of specificity in the UEAI–glycodelin staining. However, this is unlikely as staining was absent both in blood vessels showing very strong UEAI reactivity (but no glycodelin staining) and in normal secretory endometrium samples containing high level of glycodelin.

The staining of UEAI–glycodelin appeared not to be related to tumor aggressiveness because significant associations with grade, stage, size, or lymphovascular invasion were not observed. An earlier study has shown that glycodelin levels are elevated in plasma and uterine flushings in postmenopausal women with endometrial adenocarcinoma, as compared with those without neoplasia [61]. Since UEAI-reactive glycodelin was found, with one exception, only in carcinoma samples, it is likely that its circulating levels would be more specific for detection of endometrial cancer than those of total glycodelin. However, whether UEAI–glycodelin, as an apparent neoantigen in endometrial carcinoma, is a suitable marker for cancer detection, or target for vaccination strategies [62] or cancer treatment, remains to be studied.

In conclusion, using mass spectrometry glycomics strategies and lectins, we found that glycodelin expressed in HEC-1B human endometrial carcinoma cell line is differentially glycosylated as compared with normal decidualized endometrium-derived glycodelin. Despite of the differences in glycosylation these glycodelin isoforms had similar inhibitory activity on trophoblast cell invasion and PBMC proliferation, activities that we have previously found to be associated with specific glycosylation of glycodelin. We, furthermore, established a novel in situ proximity ligation assay with a lectin and an antibody, and showed that glycodelin is differentially glycosylated in endometrial carcinoma tissues as compared with normal endometrium. Whether specific detection of such glycodelin in endometrial carcinoma has any clinical significance remains to be established. Similar approach and methods can be used for studies of other glycoproteins.

## Supplementary information

Supplementary Figure
