# Supplementary material for: Altered glycosylation of glycodelin in endometrial carcinoma
Source: Lab Invest. 2020 Mar 23;100(7):1014–25. doi: 10.1038/s41374-020-0411-x (PMC7312397; doi:10.1038/s41374-020-0411-x)
Supplement: Supplementary file 1 — Supplementary Figure [file 41374_2020_411_MOESM1_ESM.pdf]

Supplementary Figure

MALDI-TOF mass spectrum of N-glycans obtained from glycodelin expressed by HEC-1B. Regions from the MALDI-MS spectrum from Figure 1 are expanded for clarity and to show the complexity of the glycome. To ease comparison, the mass regions presented here are similar as in previous publications (5,7,9). The N-glycans were released by PNGase F and permethylated ("Materials and Methods"). Data were acquired in the positive ion mode $[M+Na]^+$. Peak assignments are based on ^{12}C isotopic composition together with knowledge of the biosynthetic pathways, and some of the structures were confirmed by MS/MS. The sugar symbols are simplified representation of glycan structures and are explained in a key box on each page. Sugars shown on top of the bracket have not had their antenna location unequivocally defined. For simplicity, specific linkages are not assigned in the mass spectra and only one branching pattern for tri-antennary structures is shown. Therefore, the position of an antenna in a cartoon does not imply designation of a specific arm. Peaks which are labelled with an "x" are due to known contaminants. “New” indicates major glycans not previously reported to be present in glycodelin-A (5,7,9).

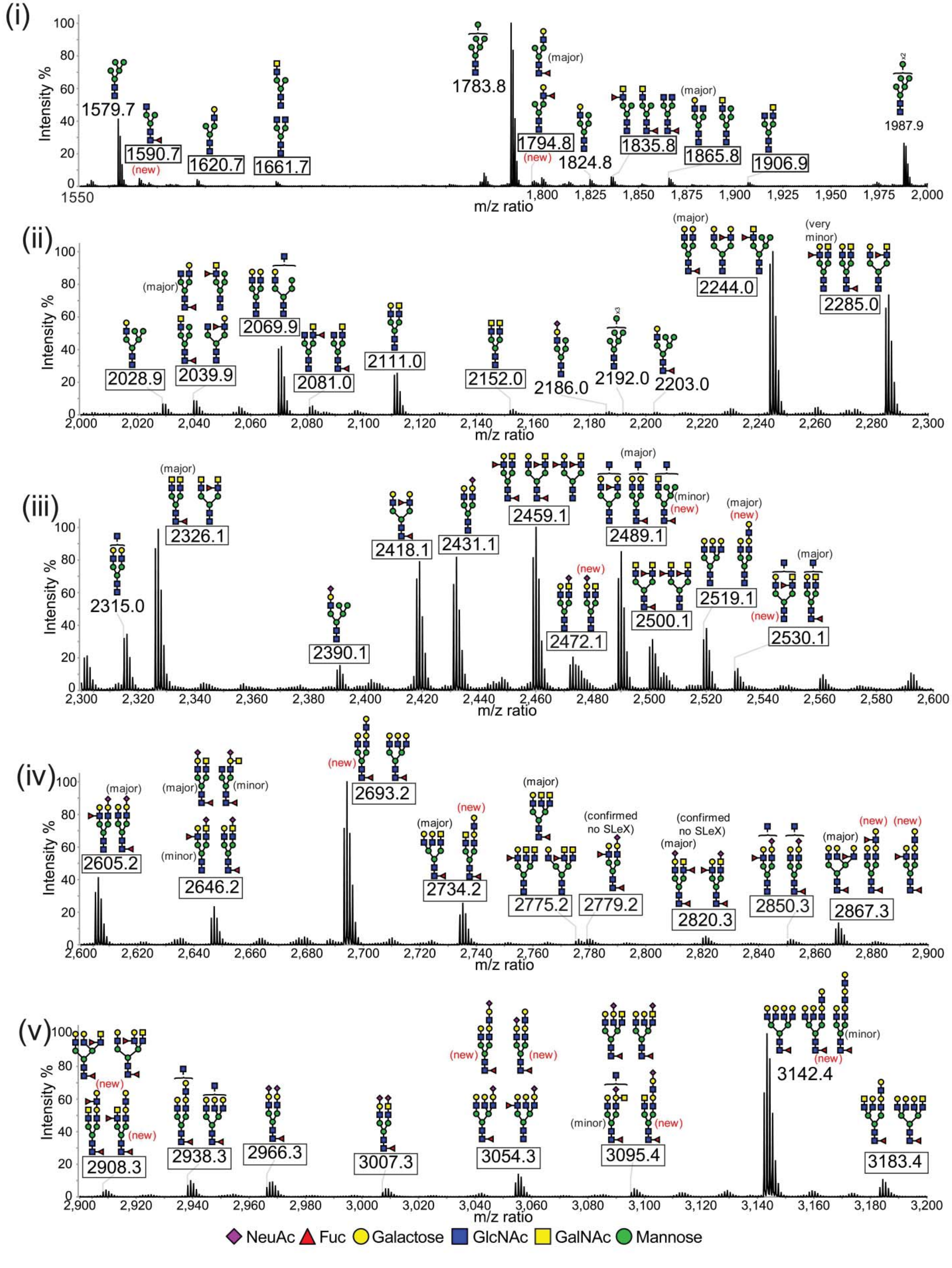

(i)
(ii)
(iii)
(iv)
(v)
Intensity %
m/z ratio
1579.7
1590.7
(new)
1620.7
1661.7
1783.8
1794.8
1824.8
1835.8
1865.8
1906.9
1987.9
2028.9
2039.9
2069.9
2081.0
2111.0
2152.0
2186.0
2192.0
2203.0
2244.0
2285.0
(very minor)
2315.0
2326.1
2390.1
2418.1
2431.1
2459.1
2472.1
2489.1
2500.1
2519.1
2530.1
2605.2
2646.2
2693.2
2734.2
2775.2
2779.2
2820.3
2850.3
2867.3
(confirmed no SLeX)
2908.3
2938.3
2966.3
3007.3
3054.3
3095.4
3142.4
3183.4
(major)
(minor)
NeuAc Fuc Galactose GlcNAc GalNAc Mannose

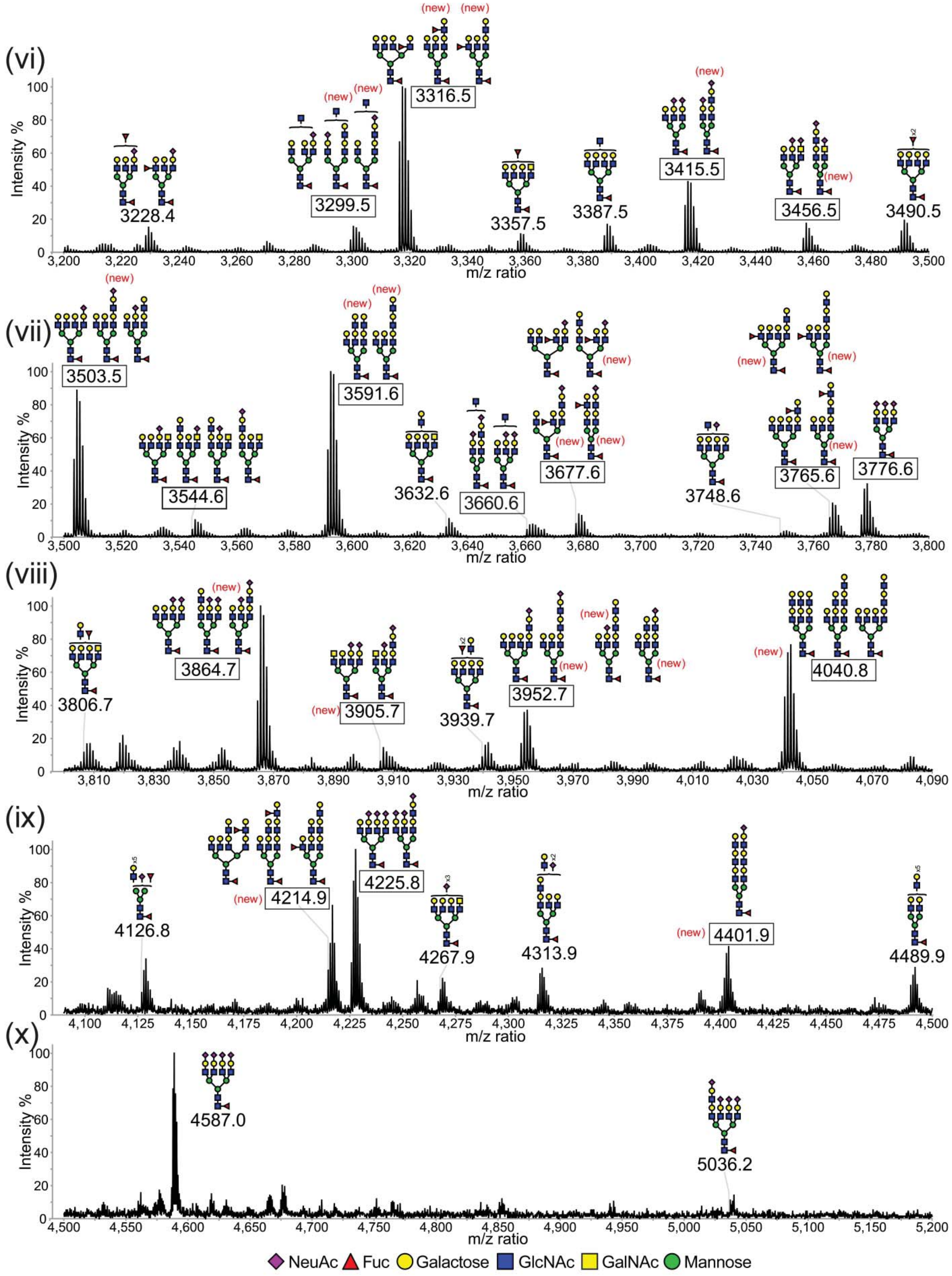
(vi)
3228.4
3299.5
3316.5
3357.5
3387.5
3415.5
3456.5
3490.5
(vii)
3503.5
3544.6
3591.6
3632.6
3660.6
3677.6
3748.6
3765.6
3776.6
(viii)
3806.7
3864.7
3905.7
3939.7
3952.7
4040.8
(ix)
4126.8
4214.9
4225.8
4267.9
4313.9
4401.9
4489.9
(x)
4587.0
5036.2
(new)
Intensity %
m/z ratio
NeuAc Fuc Galactose GlcNAc GalNAc Mannose
